# Syphilitic Ulcers

**Published:** 1871-12

**Authors:** 


Iodoform Ointment for Syphilitic Ulcers.—In the Boston City Hospital iodoform ointment, in connection with iodide of potassium, is extensively and successfully used in the treatment of syphilitic ulcers and rupia. Dr. W. Ingalls, attending surgeon, advocates this formula in two obstinate cases under his care: ℞ Iodoformi, ʒss.; Spts. vini rect., q. s.; Adipis suill., ʒ viiss. M.—*Boston Med. and Surg. Journal.*

